# Postoperative adjuvant TACE for patients of hepatocellular carcinoma in AJCC stage I: friend or foe? a propensity score analysis

**DOI:** 10.18632/oncotarget.15793

**Published:** 2017-03-01

**Authors:** Yifan Tong, Zheyong Li, Yuelong Liang, Hong Yu, Xiao Liang, Hui Liu, Xiujun Cai

**Affiliations:** ^1^ Medical College of Zhejiang University, Hangzhou, China; ^2^ Department of General Surgery, Sir Run Run Shaw Hospital Affiliated to Medical College of Zhejiang University, Hangzhou, China; ^3^ Department of Biological Treatment Research Center, Sir Run Run Shaw Hospital Affiliated to Medical College of Zhejiang University, Hangzhou, China

**Keywords:** TACE, overall survival, disease-free survival, hepatocellular carcinoma, AFP

## Abstract

**Background:**

Although the transcatheter arterial chemoembolization (TACE) was demonstrated to be an alternative treatment of hepatocellular carcinoma with favorable oncological effect, the benefit of postoperative adjuvant TACE was still controversial. The aim of this study was to evaluate the effect of postoperative TACE in hepatocellular carcinoma.

**Results:**

The 1, 3, and 5–year overall and disease–free survival rates were comparable between Surgery+TACE and Surgery groups. In subgroup analysis, tumor size (≥ 5 cm) was detrimental to disease–free survival (*p* = 0.028) and an inferior tendency of overall survival was presented. Besides, repeated TACE for patients contributed to a poor disease–free survival (*p* = 0.005). While, postoperative adjuvant TACE improved the overall survival in patients with high preoperative alpha–fetoprotein or positive pathologically (*p* = 0.039 and *p* = 0.045).

**Materials and Methods:**

The data were collected from consecutive patients between January 2010 and September 2014. After propensity score matching, baseline characteristics, overall and disease–free survival were compared between two groups. Subsequently, univariate and subgroup analysis were carried on.

**Conclusions:**

Our study indicated that single postoperative adjuvant TACE was beneficial for selected patients of stage I with tumor less than 5 cm, or high preoperative alpha–fetoprotein in serum or positive of alpha–fetoprotein pathologically.

## INTRODUCTION

Hepatocellular carcinoma (HCC) is the fifth common cancer and the third leading cause of cancer–related death worldwide. [1] Merely 20% patients diagnosed as HCC are applicable for operation, and transcatheter arterial chemoembolization (TACE) is the refenrenced treatment for patients with unresectable tumor. [2–5] Theoretically, TACE is not only inducing the hypoxia and necrosis of tumor by embolism of feeding artery, but also suppressing tumor activity through chemotherapy. [6, 7]

While, it is worrying that postoperative TACE, as an adjuvant therapy, brings down therapeutic effect due to the removal of tumor previously. Meanwhile, postoperative TACE increases the adverse events. In some medical centers, especially in Asia, postoperative adjuvant TACE was recommended experientially for patients with adverse prognostic factors (e.g. thrombus of portal vein, microvascular invasion, multiple lesions). However, only few of studies with respect to postoperative adjuvant TACE in the treatment of HCC in American Joint Committee On Cancer (AJCC) stage I, were published, [8–11]. Therefor, the postoperative adjuvant TACE, as a friend or foe, needs to be explored in the comprehensive treatment of HCC in AJCC stage I.

Although the randomized controlled trial is the ideal method to elucidate the benefits and drawbacks of postoperative adjuvant TACE, it is difficult to recruit enough patients to analyze the survival rates. Under such a circumstance, retrospective study based on propensity score matching minimized the selection bias to increase the level of evidence. The aim of this study was to analyze retrospectively whether the postoperative adjuvant TACE was beneficial or not for patients of HCC in AJCC Stage I.

## RESULTS

Each group contained 83 patients. Although some original baseline characteristics presented the heterogeneity (Supplementary Table 1), all demographic characteristics of two groups shown in Table 1 were comparable after propensity score matching. Table 2 compared the surgical and pathological outcomes of two groups, which presented similar results without significant difference. 37 out of 83 patients in Surgery+TACE group underwent selected TACE. Besides, 19 (22.9%) patients with 27 adverse events occurred totally, and all patients above–mentioned recovered smoothly with symptomatic treatments.

**Table 1:** Demographic characteristics

	Surgery Group (*n* = 83)	Surgery+TACE Group (*n* = 83)	*P*–value
Age (years)	61.0 (36–83)	57.0 (34–83)	0.121
Gender (Male)	71 (85.5%)	72 (86.7%)	0.822
BMI (kg/m^2^)	23.9 (17.5–31.6)	24.1 (16.2–32.2)	0.612
Serum HBV	64 (77.1%)	70 (84.3%)	0.157
Serum HCV	1 (1.2%)	0 (0%)	1.000
Hemoglobin (g/L)	14.0 (8.0–17.1)	14.3 (8.9–17.1)	0.125
ALT (IU/L)	38.0 (7.0–128.0)	36.0 (13.0–186.0)	0.092
Prothrombin Time (s)	13.4 (11.5–17.3)	13.5 (11.7–23.0)	0.696
Albumin (g/L)	40.5 (28.2–51.0)	41.8 (23.4–50.6)	0.298
Total Bilirubin (μmol/L)	15.5 (3.2–50.7)	14.8 (3.4–47.9)	0.451
Alpha–fetoprotein (ng/mL)	14.7 (1.5–11408.0)	22.8 (1.5–50500.0)	0.743
Child–Pugh Grade			1.000
A	80 (96.4%)	81 (97.6%)	
B	3 (3.6%)	2 (2.4%)	
Cirrhosis	36 (43.4%)	35 (42.2%)	0.875
HBV–related	32 (38.6%)	33 (39.8%)	0.874
HCV–related	1 (1.2%)	0 (0%)	1.000
Schistosome	3 (3.6%)	1 (1.2%)	0.620
Alcoholic	0 (0%)	1 (1.2%)	1.000
Family History of HCC	8 (9.6%)	13 (15.6%)	0.243

Values are presented as median (range) or number (percentage). TACE, Transcatheter Arterial Chemoembolization; BMI, Body Mass Index; ALT, Alanine Aminotransferase; HBV, Hepatitis B virus; HCV, Hepatitis C virus; HCC, Hepatocellular Carcinoma.

**Table 2:** Short-term and pathological outcomes

	Surgery Group (*n* = 83)	Surgery+TACE Group (*n* = 83)	*P*-value
Tumor Location			
I	3 (3.6%)	0 (0%)	0.245
II	7 (8.4%)	6 (7.2%)	0.773
III	6 (7.2%)	8 (10.6%)	0.576
IV	15 (18.1%)	11 (13.3%)	0.393
V	17 (20.5%)	14 (16.9%)	0.550
VI	21 (25.3%)	29 (34.9%)	0.176
VII	18 (21.7%)	20 (24.1%)	0.712
VIII	14 (16.9%)	22 (26.5%)	0.132
Maximum Tumor Diameter (cm)	3.0 (0.9–11.0)	3.8 (0.7–11.6)	0.242
Magnitude of Operation			0.417
Major	9 (10.8%)	6 (7.2%)	
Minor	74 (89.2%)	77 (92.8%)	
Extent of resection			
Wedge Resection	35 (42.6%)	37 (44.6%)	0.754
Segmentectomy	17 (20.5%)	12 (14.5%)	0.307
Left Lateral Lobectomy	11 (13.3%)	11 (13.3%)	1.000
Right Posterior Lobectomy	4 (4.8%)	6 (7.2%)	0.744
Anatomical Segmentectomy	7 (8.4%)	11 (13.3%)	0.318
Left Hepatectomy	3 (3.6%)	3 (3.6%)	1.000
Right Hepatectomy	6 (7.2%)	3 (3.6%)	0.493
Satellite Focus	1 (1.2%)	6 (7.2%)	0.122
Capsule	7 (8.4%)	5 (6.0%)	0.549
Ruptured	1 (1.2%)	2 (2.4%)	1.000
TACE			
Selected	-	37 (44.6%)	
Non-selected	-	46 (55.4%)	
Adverse Events			
Fever (> 38.0°C)	-	3 (3.6%)	
Abdonimal Pain	-	8 (10.6%)	
Nausea or Vomiting	-	10 (12.0%)	
Thrombocytopenia (< 5 × 10^9^/L)	-	2 (2.4%)	
Leukocytopenia (< 1 × 10^9^/L)	-	4 (4.8%)	
Hepatic Failure	-	0 (0%)	

Values are presented as median (range) or number (percentage). TACE, Transcatheter Arterial Chemoembolization; Major resection is defined as equal or more than three segmentectomy.

**Figure 1:**
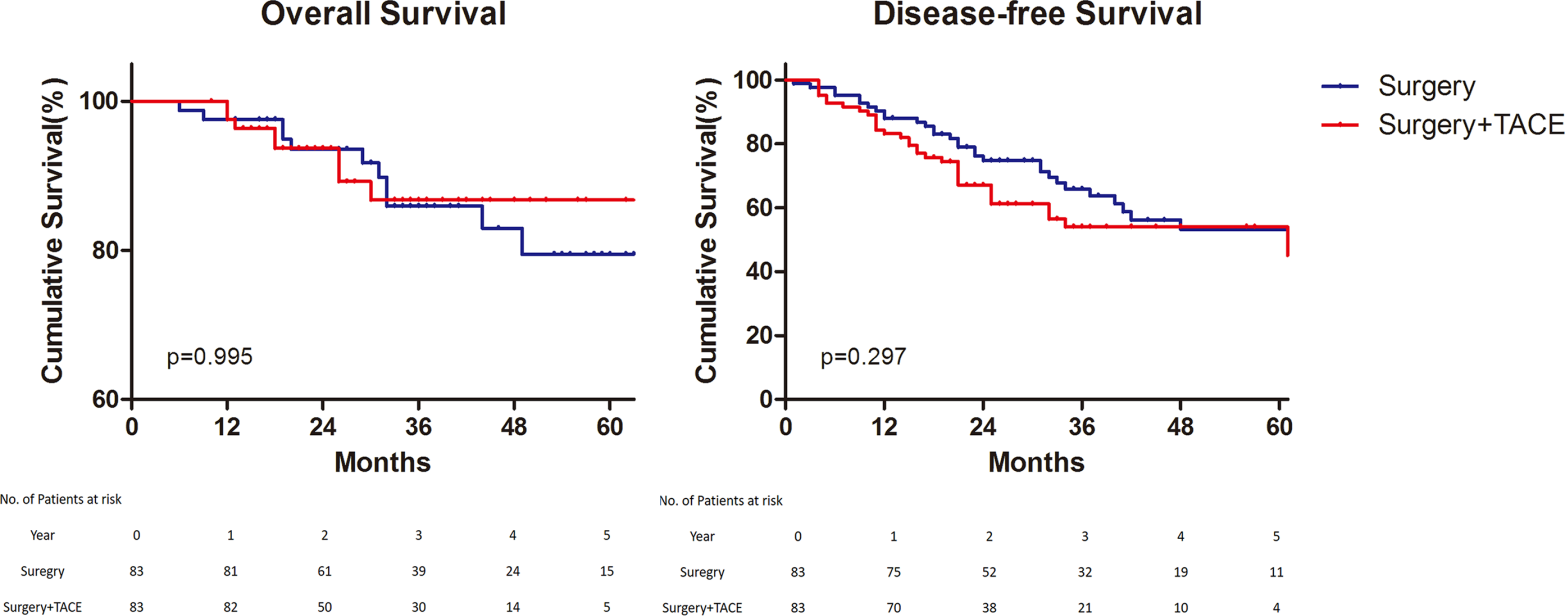
Overall and disease–free survival Overall survival (Left) and Disease–free survival (Right). TACE, Transcatheter Arterial chemoembolization.

For long–term outcomes, the mean time of follow–up were 37.6 months in Surgery group and 31.5 months in Surgery+TACE group. The 1, 3 and 5–year overall survival rates were 97.6%, 84.8%, and 78.2%, respectively, in the Surgery group, and 100%, 91.5%, and 83.3%, respectively, in the Surgery+TACE group (*p* = 0.995). The 1, 3 and 5–year disease–free survival rates were 90.4%, 64.3%, and 51.6%, respectively, in the Surgery group, and 87.5%, 56.4%, and 50.0%, respectively, in the Surgery+TACE group (*p* = 0.297) (Figure 1).

**Figure 2:**
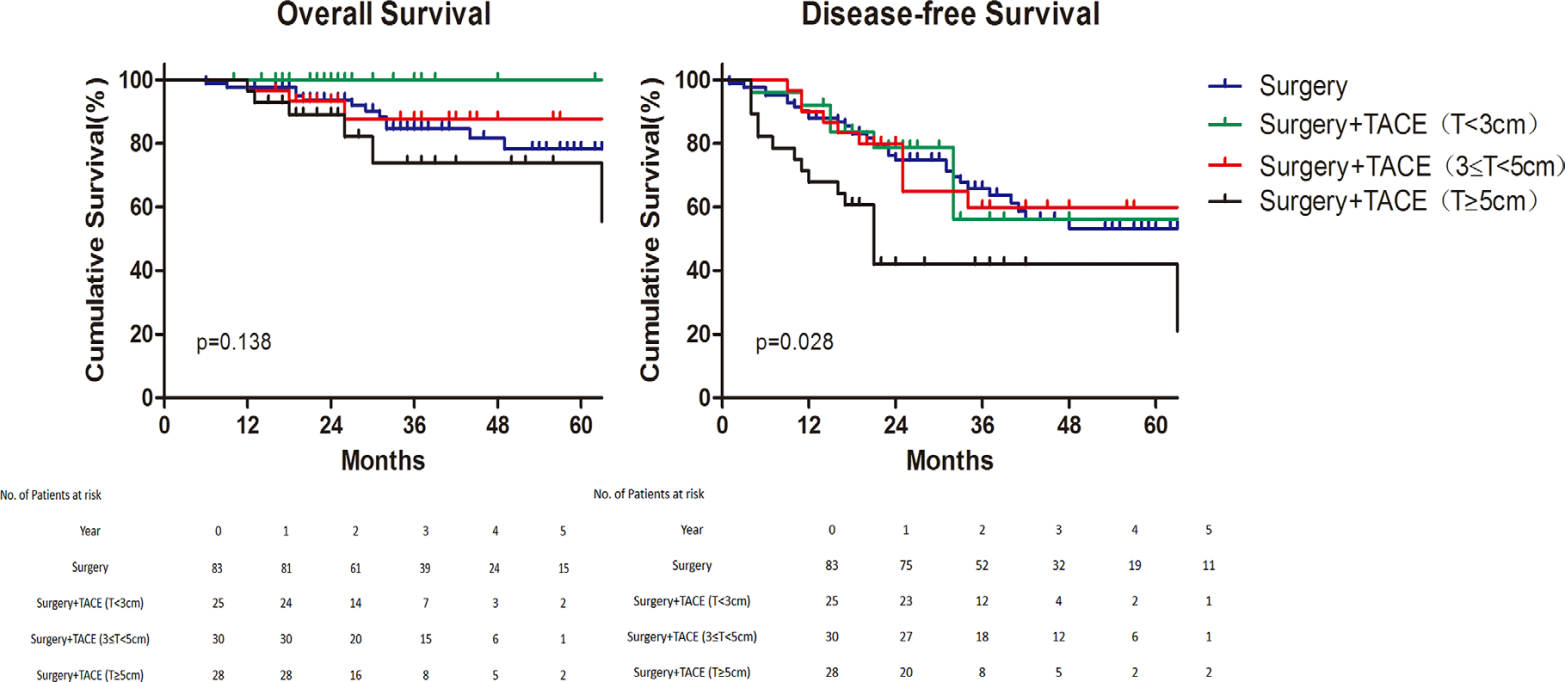
Subgroup analysis of survival for different tumor size Overall survival (Left) and Disease–free survival (Right). TACE, Transcatheter Arterial chemoembolization.

Subsequently, univariate analysis indicated that satellite focus and rupture of tumor were shown as negative factors for overall and disease–free survival (Supplementary Figure 1). While tumor size (≥ 5 cm) was an independent adverse factor for disease–free survival with hazard ratio of 1.73 (*p* = 0.013). Besides, not only detrimental effect was shown in disease–free survival (*p* = 0.028), an inferior tendency of overall survival among the patients with large tumor size (≥ 5 cm) was presented although without significant difference (Figure 2).

In subgroup analysis, patients of Surgery+TACE group started to receive TACE from half a month to eight months after liver resection (two patients delayed the TACE procedure in six and eight months as personal reasons), and the mean of times of TACE was 1.3 ± 0.6 times. With respect to the timing of adjuvant TACE (the boundary was 60–day after operation), earlier TACE did not improve the prognosis. Furthermore, repeated TACE for patients was detrimental to disease–free survival (*p* = 0.005) (Supplementary Figure 2). Compared with patients underwent non–selected TACE, selected TACE group presented a better tendency of overall survival but without significant difference, while the disease–free survival seemed paralleled (Supplementary Figure 3). And the adverse events of two groups were similar.

**Figure 3:**
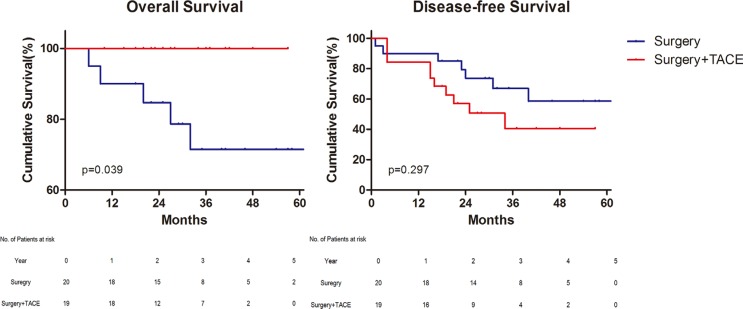
Survival for patients with preoperative alpha–fetoprotein ≥ 200 ng/mL Overall survival (Left) and Disease–free survival (Right). TACE, Transcatheter Arterial chemoembolization.

**Figure 4:**
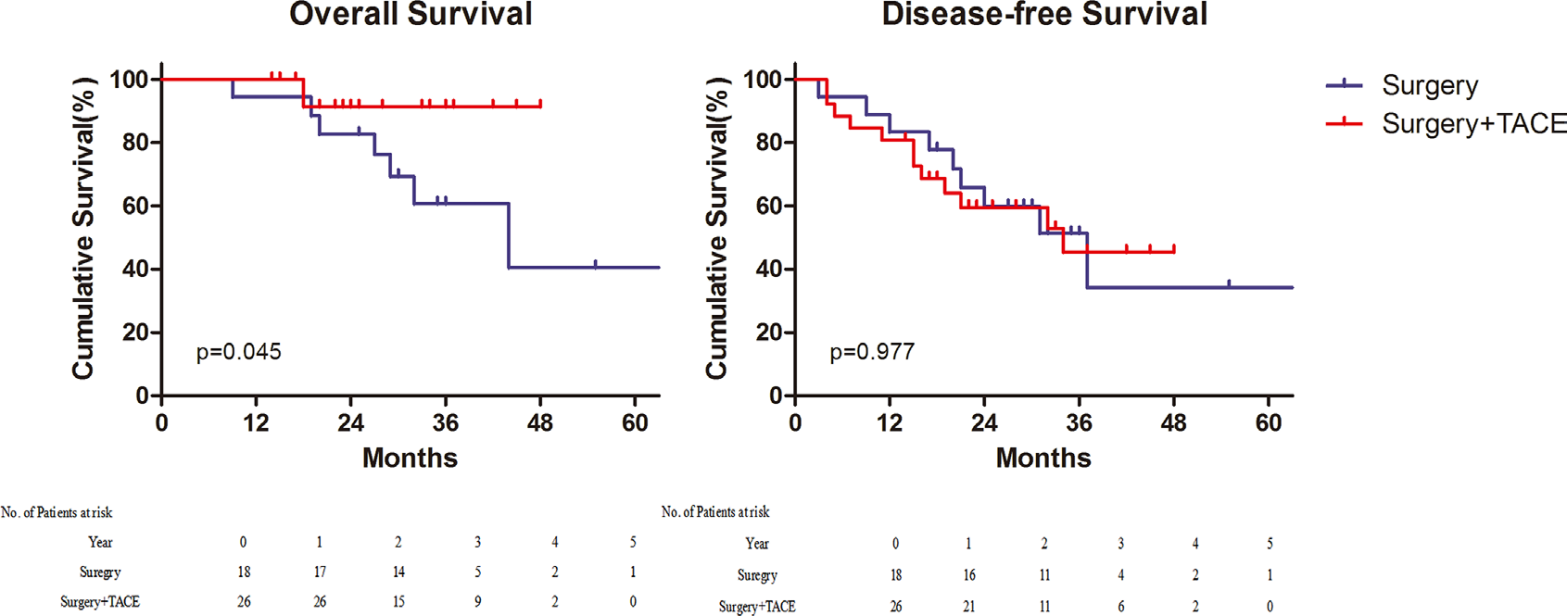
Survival for patients with immunohistochemical alpha–fetoprotein positive Overall survival (Left) and Disease–free survival (Right). TACE, Transcatheter Arterial chemoembolization.

Notwithstanding, the AFP did not display the correlation with prognosis in univariate analysis before, it was yet demonstrated to have an impact on survival. For patients with high preoperative AFP (≥ 200 ng/mL) in peripheral serum, postoperative TACE improved the overall survival (*p* = 0.039) (Figure 3). On the contrary, in patients with low peripheral AFP (< 200 ng/mL) before operation, both the overall and disease–free survival of groups were comparable (Supplementary Figure 4). In terms of expression of AFP in the specimen, 44(26.5%) patients were positive, 68 (41.0%) were negative, and 54 (32.5%) were undetected. According to the analysis of survival, AFP positive of immunohistochemistry was also the indication of postoperative TACE (Figure 4) (Supplementary Figure 4).

## DISCUSSION

Globally, TACE was the preferential treatment for unresectable HCC and an alternative treatment for small or intermediate HCC with favorable outcomes, although the adverse effects, extra cost, lack of high–level evidence to support the benefit of prognosis from postoperative TACE, limited its application in clinical. [12–20] For the adjuvant TACE, the absence of tumor contributed to a complicated influence by changing the micro–environment of tumor, although the concentration of chemotherapy drugs were increased through the embolism. The hypoxia effect caused by TACE procedure, also led to the ischemia of tumor–free hepatocytes, stimulating the expression of vascular endothelial growth factor (VEGF) and activation of related signaling pathways, which induced the neoangiogenesis and formed tumor favorite micro–environment. [12, 21–24] Besides residual tumor cell became aggressive though up–regulation of the VEGF, the susceptibility to chemotherapy declined as the expression of drug–resistant genes increased on hypoxia stress, which might be partial responsible for the negative impact on prognosis.

In the present study, both the overall and disease–free survival of Surgery+TACE and Surgery group were comparable. According to prognostic analysis with cox regression, tumor size, satellite focus, and rupture of tumor were adverse prognostic factors. To speak of patients with satellite focus or rupture of tumor, the small sample size of positive cases restricted a further analysis. As tumor size was confirmed to be relevant to disease–free survival, large tumor was apt to s necrosis and releasing VEGF especially in the center area. A Possible hypothesis was that postoperative adjuvant TACE triggered the proliferation and activity of residual cancer cells, which overrode the anti–tumor effect under the condition of hypoxia. Definitely, the mechanism remained vague and should be illustrated in detail.

Furthermore, molecular markers might be potential value of application in prediction and treatment of malignancies. In subgroup analysis, AFP before operation in peripheral serum as well as expression of AFP with immunohistochemistry confirmed, were demonstrated to be indications for postoperative adjuvant TACE. As detectable AFP reflected the activity of tumor. dual positive of AFP in plasma and tissue might improve the sensitivity and specificity, in evaluating the efficiency of postoperative adjuvant TACE theoretically. [25]

Although TACE compared with surgery, radiofrequency ablation, target therapy, supportive treatment were reported before, [4, 12, 14] the timing and times of TACE as an adjuvant therapy remained indistinct. Too short interval time after operation increased the risk of liver damage even hepatic failure. On the other side, the cumulative rate of recurrence or metastasis would be elevated during the overlong interval time. In our study, the TACE of later group did not show any discrepancy of prognosis in comparison with early group, while repeated TACE decreased the disease–free survival. Briefly, the optimum timing of postoperative adjuvant TACE needed to be further studied and repeated TACE was not recommended. And selected TACE presented a better tendency of overall survival compared with non–selected TACE and Surgery groups. However, much effort was needed to verify the advantage of selected TACE in the reduction of side effects andnon–inferior oncological efficacy.

Several limitations of this study should be acknowledged. To begin with, potential bias could not be completely eliminated as a retrospective study. The propensity score matching was applied to reduce the selective bias as possible as we could. Additionally, the differentiation of tumor was not included in the survival analysis due to a large proportion of missing data. There was evidence that inclusion of variables with large proportion of missing data might introduce bias, though techniques handling missing values are becoming more and more sophisticated. [26] Eventually, in terms of AFP for prognosis, the synergy effect or connection inside, if existed, showed a terrific potential of application clinically. The dual positive of AFP expression in serum and tissues was more likely to improve the sensitivity and specificity in the prognostic model of survival. However, a part of patients without detecting the expression of AFP pathologically, decreased the validity of AFP on prognosis.

In conclusion, our study by propensity score matching indicated that, single adjuvant TACE after hepatectomy with negative margin was beneficial for patients of AJCC stage I with tumor less than 5cm, or high preoperative AFP or positive of AFP in specimen. Further investigations are definitely warranted.

## MATERIALS AND METHODS

### Study design

This study was approved by the Ethical Committees for Human Subjects at Sir Run Run Shaw Hospital affiliated to Zhejiang University, China. Data of consecutive patients with written informed consent, who underwent hepatectomy from January 2010 to September 2014 were collected retrospectively.

The patients with hepatocellular carcinoma in AJCC stage I confirmed pathologically were eligible for this study. The selection criteria for postoperative adjuvant TACE were good general condition, Child A or B liver function. And the patients with positive margin microscopically or palliative liver resection; transcatheter artery chemotherapy, preoperative adjuvant therapies (e.g. preoperative TACE, radiofrequency ablation, percutaneous ethanol injection, chemotherapy or sorafenib), or other postoperative adjuvant therapies expect TACE before recurrence and metastasis during the period of follow–up, were excluded.

Patients enrolled who underwent surgery followed by adjuvant TACE were defined as Surgery+TACE group, while who underwent hepatectomy alone without adjuvant treatment were defined as Surgery group. From 115 patients of Surgery+TACE group and 91 patients of Surgery group, 83 pairs were matched by propensity score analysis. Demographic characteristics, surgical and pathological outcomes, and long–term outcomes including overall survival and disease–free survival, were compared between two groups.

### TACE procedure and follow–up

All patients underwent TACE procedure by experienced interventional radiologists of same team. The 5–F French catheter was access to the aorta abdominalis through superficial femoral artery with the seldinger technique, followed by hepatic arterial angiography with guidance of Yashiro catheter. As all patients underwent hepatectomy, appropriate arteries or branches were targeted according to the different extent of resection. Selected TACE was defined as targeted to the segment of artery. Generally, a mixture of mitomycin 10 mg, epirubicin 50 mg and lipiodol 10 ml was injected, simultaneously, oxaliplatin 150 mg was perfused intermittently, followed by embolism with spongel particles until blood flow almost stopped. Fluid–supplement therapy was performed routinely, and liver protection drugs or antibiotics were supplied, if necessary.

A standardized follow–up protocol was adopted for all the patients. During the follow–up, in addition to a detailed history taking and physical examination, the patients received ultrasound of the abdomen, serum AFP, blood routine and liver function. Abdominal Computed Tomography or Magnetic Resonance Imaging were performed when clinically indicated. Patients in Surgery+TACE group underwent first TACE were required in 15 to 90 days after operation generally. If AFP in peripheral serum was elevated, or suspicious lesion on imaging examination, or iodized oil accumulation, the repeated TACE procedure was performed. All patients of both groups were regularly followed up every 3 to 6 months until the fifth year. Later, follow–up annually was carried on.

### Statistical analysis

The continuous variables were expressed as median with range and categorical data were presented as number with proportion. Correspondingly, the Mann–Whitney *U* test was used to compare continuous variables, while the Chi–square test or Fisher exact test, was used to compare categorical variables as appropriate. Cumulative survival was presented as Kaplan–Meier curves and compared by the log–rank test. Overall and disease–free survival were analyzed with univariate cox regression model. The propensity score matching was applied to eliminate bias of selection.

**Figure 5:**
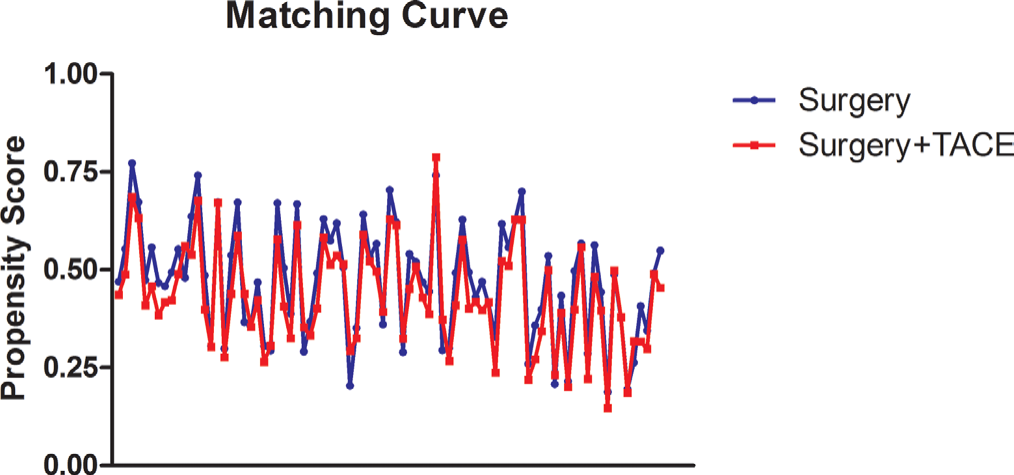
Propensity matching curve TACE, Transcatheter Arterial chemoembolization.

According to the baseline characteristics, including age, gender, body mass index (BMI), Child–Pugh grade, cirrhosis and tumor size, two groups were paired with ratio of one–to–one nearest neighbor matching estimated propensity score within 0.1 of caliper width (Figure 5). The *p*–value with two–tailed less than 0.05 was defined as significant difference. Statistical analysis was performed using SPSS, version 22.0 for Windows (IBM Corporation, Armonk, NY).

## SUPPLEMENTARY MATERIALS FIGURES AND TABLE



## Abbreviations

AFP: Alpha–fetoprotein
AJCC: American Joint Committee on Cancer
DFS: Disease–free Survival
HCC: Hepatocellular Carcinoma
OS: Overall Survival
TACE: Transcatheter Arterial chemoembolization
VEGF: Vascular Endothelial Growth Factor
