# Supplementary material for: Postoperative adjuvant TACE for patients of hepatocellular carcinoma in AJCC stage I: friend or foe? a propensity score analysis
Source: Oncotarget. 2017 Mar 1;8(16):26671–8. doi: 10.18632/oncotarget.15793 (PMC5432288; doi:10.18632/oncotarget.15793)
Supplement: Supplementary file 1 [file oncotarget-08-26671-s001.pdf]

# Postoperative adjuvant TACE for patients of hepatocellular carcinoma in AJCC stage I: friend or foe? a propensity score analysis

## Supplementary Materials

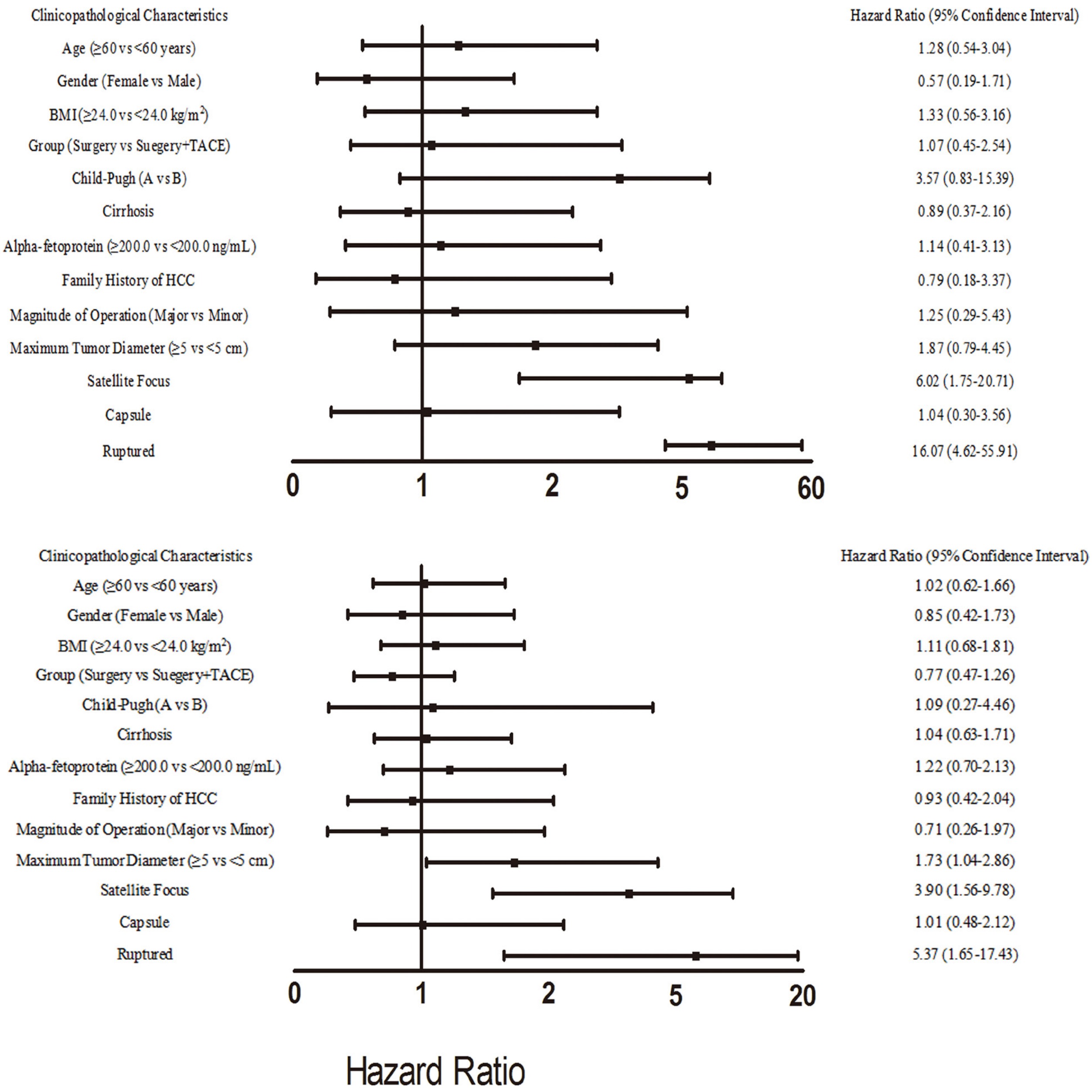


**Supplementary Figure 1: Univariate analysis of survival with cox regression model.** TACE: Transcatheter Arterial chemoembolization; BMI, Body Mass Index; HCC, Hepatocellular Carcinoma; Major resection is defined as equal or more than three segmentectomy; HR, Hazard Ratio.

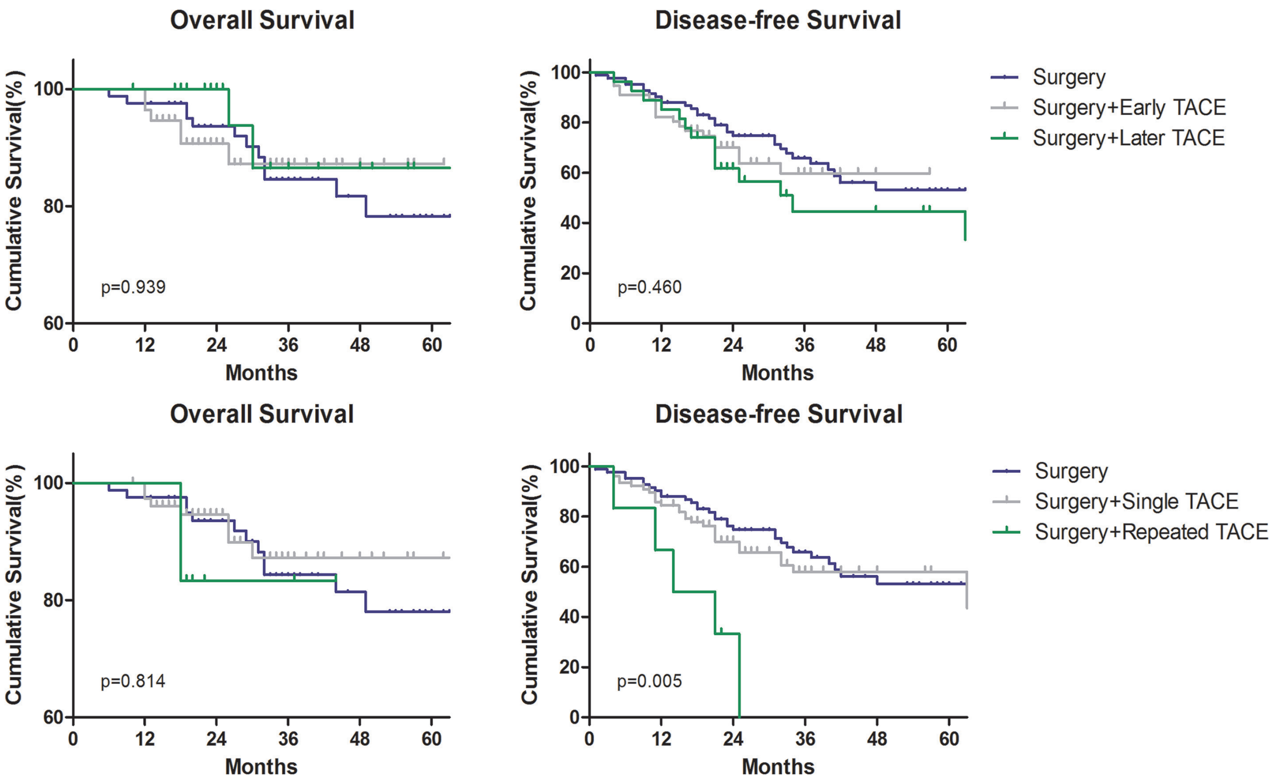


**Supplementary Figure 2: Subgroup analysis of survival with timing and times of TACE.** Overall survival (Upper left) and Disease-free survival (Upper right) for Timing of TACE. Overall survival (Lower left) and Disease-free survival (Lower right) for Times of TACE. TACE, Transcatheter Arterial chemoembolization.

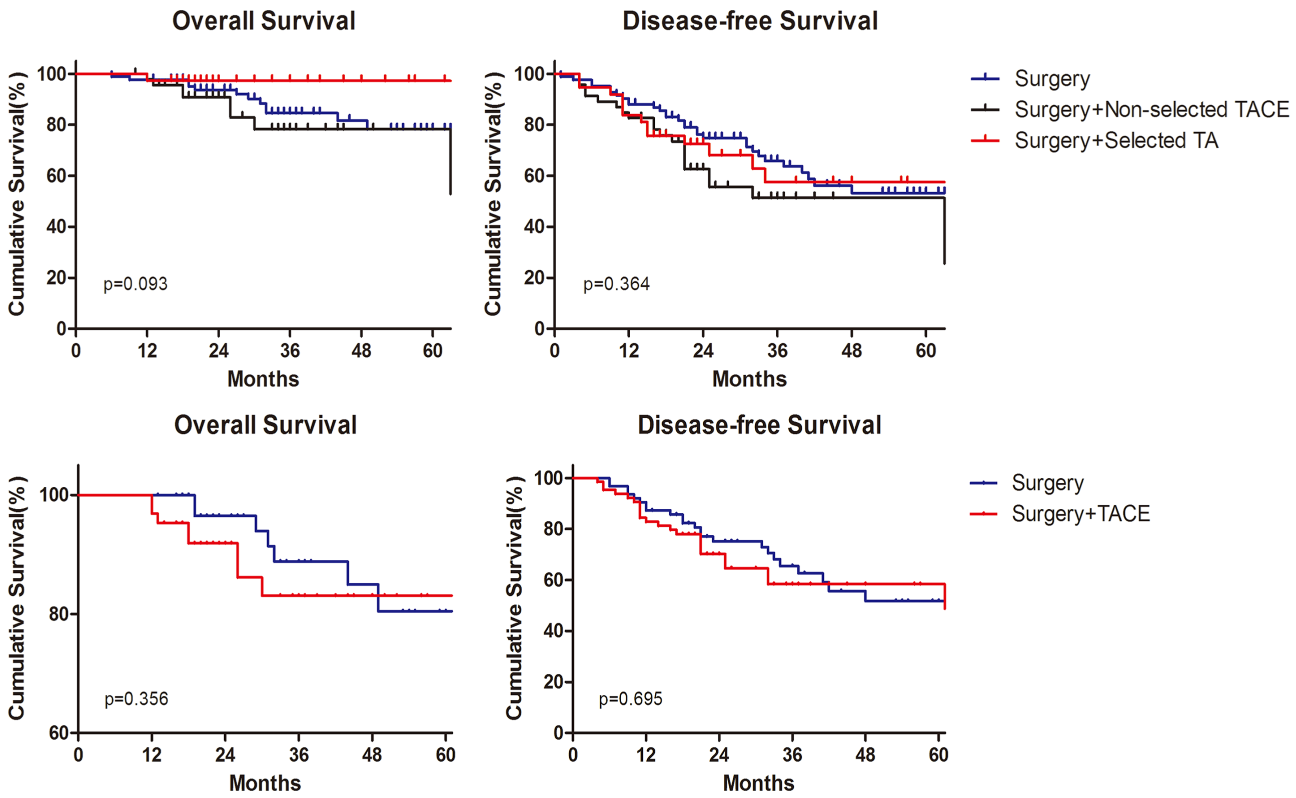


**Supplementary Figure 3: Subgroup analysis for patients with selected or Non-selected TACE and preoperative alpha-fetoprotein < 200 ng/mL.** Overall survival (Upper left) and Disease-free survival (Upper right) for patients with selected or non-selected TACE. Overall survival (Lower left) and Disease-free survival (Lower right) for patients with preoperative alpha-fetoprotein < 200 ng/mL. TACE, Transcatheter Arterial chemoembolization.

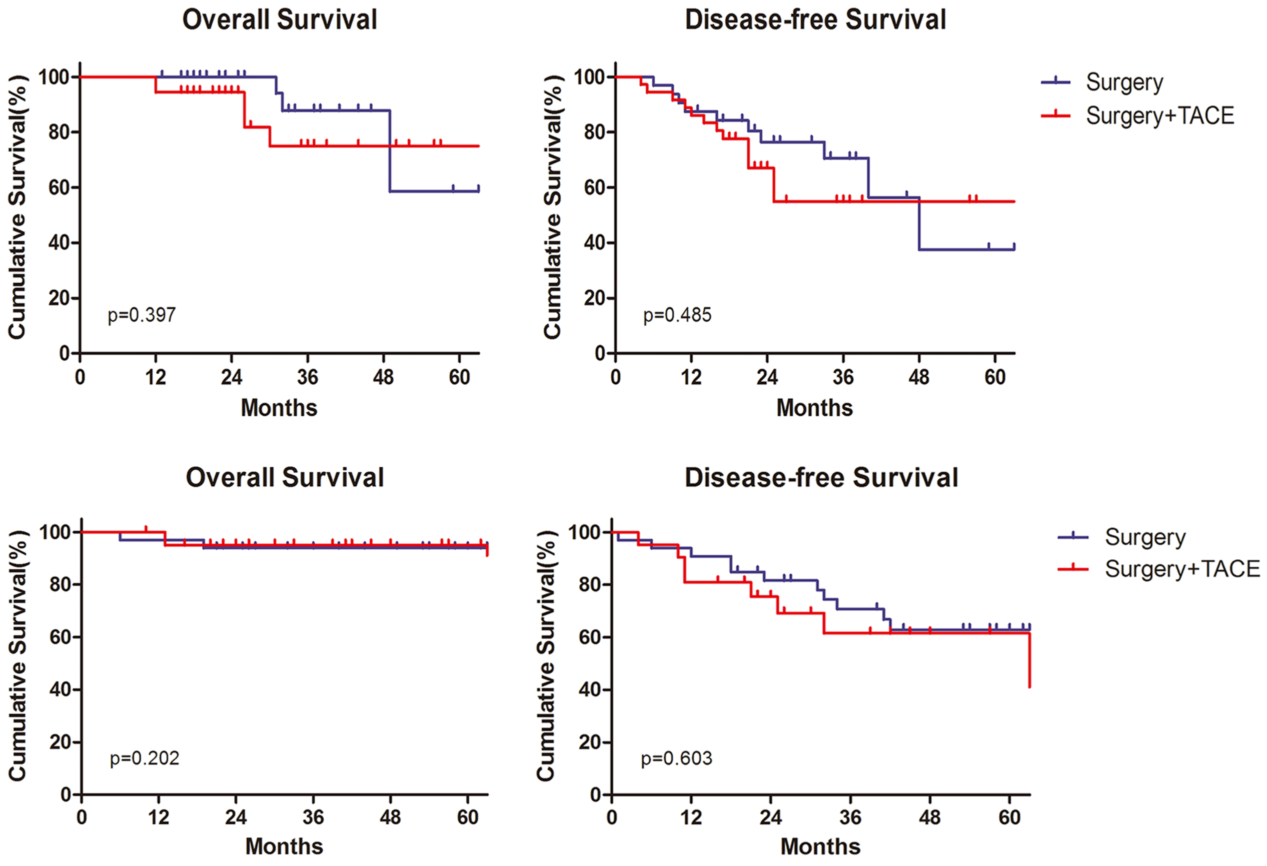


**Supplementary Figure 4: Subgroup analysis for patients with alpha-fetoprotein negative or undetected immunohistochemically.** Overall survival (Upper left) and Disease-free survival (Upper right) for patients with immunohistochemical alpha-fetoprotein negative. Overall survival (Lower left) and Disease-free survival (Lower right) for patients with immunohistochemical alpha-fetoprotein undetected. TACE, Transcatheter Arterial chemoembolization.

**Supplementary Table 1: Clinicopathological characteristics before propensity score matching**

| | Surgical Group ($n$ = 91) | Surgical+TACE Group ($n$ = 115) | $P$–value |
|---|---|---|---|
| Age (years) | 63.0 (36–84) | 39.0 (29–83) | < 0.001 |
| Gender (male) | 78 (85.7%) | 100 (87.0%) | 0.796 |
| BMI (kg/m$^2$) | 24.2 (17.5–31.6) | 23.2 (15.4–32.2) | 0.330 |
| Serum HBV | 67 (73.6%) | 101 (87.8%) | 0.009 |
| Serum HCV | 1 (1.1%) | 0 (0%) | 0.442 |
| Hemoglobin (g/L) | 13.9 (8.0–17.1) | 14.3 (8.9–17.1) | 0.113 |
| Prothrombin Time (s) | 13.4 (11.3–17.5) | 13.5 (11.7–23.0) | 0.639 |
| ALT (IU/L) | 28.0 (7–128) | 36.0 (5–211) | 0.015 |
| Albumin (g/L) | 39.9 (28.2–51.0) | 40.7 (23.4–50.6) | 0.234 |
| Total Bilirubin (μmol/L) | 15.4 (3.2–50.7) | 14.8 (3.4–47.9) | 0.771 |
| Alpha-fetoprotein (ng/mL) | 14.7 (1.4–14104.8) | 18.3 (1.5–50500.0) | 0.563 |
| Cirrhosis | 40 (44.0%) | 45 (39.1%) | 0.185 |
| HBV-related | 35 (38.5%) | 43 (37.4%) | 0.875 |
| HCV-related | 1 (1.1%) | 0 (0%) | 0.442 |
| Schistosome | 4 (4.4%) | 1 (0.8%) | 0.172 |
| Alcoholic | 0 (0%) | 1 (0.8%) | 1.000 |
| Child-Pugh Grade | | | 0.702 |
| A | 87 (95.6%) | 112 (97.4%) | |
| B | 4 (4.4%) | 3 (2.6%) | |
| Family History of HCC | 8 (8.8%) | 16 (13.9%) | 0.255 |
| Tumor Location | | | |
| I | 3 (3.3%) | 0 (0%) | 0.257 |
| II | 13 (14.3%) | 7 (6.1%) | 0.084 |
| III | 7 (7.7%) | 8 (7.0%) | 0.840 |
| IV | 15 (16.5%) | 13 (11.3%) | 0.281 |
| V | 17 (18.7%) | 23 (20.0%) | 0.812 |
| VI | 26 (28.6%) | 41 (35.7%) | 0.281 |
| VII | 18 (19.8%) | 30 (26.1%) | 0.288 |
| VIII | 14 (15.4%) | 30 (26.1%) | 0.063 |
| Maximum Tumor Diameter (cm) | 3.0 (0.9–11.0) | 2.0 (0.7–11.6) | 0.180 |
| Magnitude of Operation | | | 0.311 |
| Major | 9 (9.9%) | 7 (6.1%) | |
| Minor | 82 (90.1%) | 108 (93.9%) | |

Values are presented as median (range) or number (percentage). TACE: Transcatheter Arterial Chemoembolization; BMI, Body Mass Index; ALT, Alanine Aminotransferase; HBV, Hepatitis B virus; HCV, Hepatitis C virus; HCC, Hepatocellular Carcinoma. Major resection is defined as equal or more than three segmentectomy.

## Survival rate algorithm

Overall Survival Rate = (1- Mortality) × 100%
Mortality = Deaths/(No. at risk + No. of truncation/2) × 100%
Disease-free Survival Rate = (1- Recurrence Rate) × 100%
Recurrence Rate = No. of recurrence/(No. at risk + No. of truncation/2) × 100%
